# The Value of Imagistics in Early Diagnosis of Tuberous Sclerosis

**DOI:** 10.1155/2020/1309184

**Published:** 2020-03-10

**Authors:** Claudia Ioana Borțea, Vlad Laurentiu David, Florina Stoica, Cezara Mureșan, Marioara Boia

**Affiliations:** ^1^Department of Neonatology, “Victor Babes” University of Medicine and Pharmacy, Timisoara, Romania; ^2^Department of Pediatric Surgery and Orthopedics, “Victor Babes” University of Medicine and Pharmacy, Timisoara, Romania; ^3^Department of Ophthalmology, Emergency Municipal Clinical Hospital, Timisoara, Romania; ^4^Department of Obstetrics and Gynecology, “Victor Babes” University of Medicine and Pharmacy, Timisoara, Romania

## Abstract

Tuberous sclerosis complex is a multisystemic genetic disorder with high phenotypical variability. Its progress frequently brings along autism (61%), epilepsy, intellectual disability (45%), and neurocognitive impairment (Gipson and Johnston, 2017). We are considering the case of an infant suspected with tuberous sclerosis complex by imagistic investigation in the prenatal period. The pre- and postnatal ultrasound, fetal MRI, ophthalmoscopy, and dermatological and neurological examinations were used for diagnosis and follow-up. The seven major and minor criteria were regarded as sufficient for accurate diagnosis.

## 1. Introduction

Tuberous sclerosis complex (TSC) is a disorder of cellular differentiation, proliferation, and migration in early development characterized by the formation of benign, hamartomatous lesions in virtually any organ system. It is an autosomal dominant genetic disease, even if two thirds of patients are found with sporadic mutations [1]. The two involved protein-coding genes are TSC1 and TSC2, with an important role in suppressing the mammalian target of rapamycin (mTOR) pathway, influencing the cell growth and proliferation [2–4]. The mutations cannot be identified by conventional genetic tests in a significant proportion of cases (10–25%), so negative results of genetic tests do not exclude TSC. Clinical criteria continue to be the main diagnosis way, the imagistic assessment being useful in early diagnosis [5].

## 2. Case Report

**Figure 1:**
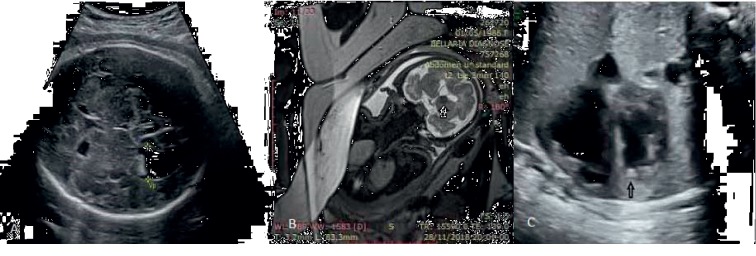
(a) Fetal ultrasound—bilateral hydrocephalus, mild on the right side and severe on the left side; (b) fetal MRI—lesion next to the foramen of Monro, typical for SEGA; (c) fetal ultrasound—echogenic intracardiac focus on the left ventricular apex.

We present the case of a 4-month-old boy, without relevant personal history, from a *gravida 6 para 5 pregnancy*, accurately monitored, with a physiological course. Bilateral hydrocephalus, severe on the left side, was found by fetal ultrasound (32 w + 5 d). The probability of syndromic context was considered low, without any other anomalies. The fetal MRI (33 w + 6 d) showed severe ventriculomegaly; periventricular lesions along the ganglionic eminence area like a subependymal nodular heterotopia, with partial calcification; a bigger lesion was located next to the left foramen of Monro typical for subependymal giant cell astrocytoma (SEGA), which can be responsible for cerebrospinal fluid disorder; an extra modification of MRI signal from the right lateral ventricle surface to the insular cortex, as a sign of transmantle cortical dysplasia. The fetal ultrasound (34 w + 4 d) showed an echogenic intracardiac tumor on the left ventricular apex, being interpreted as rhabdomyoma, due to the fetal MRI result (Figure 1).

It was a natural birth, at term, with physiological neonatal adaptation. The clinical exam at birth showed on the upper back midline a large (about 25 mm) erythematous plaque with orange-peel surface, suggestive for Shagreen patch and dermatologically confirmed (Wood's lamp).

**Figure 2:**
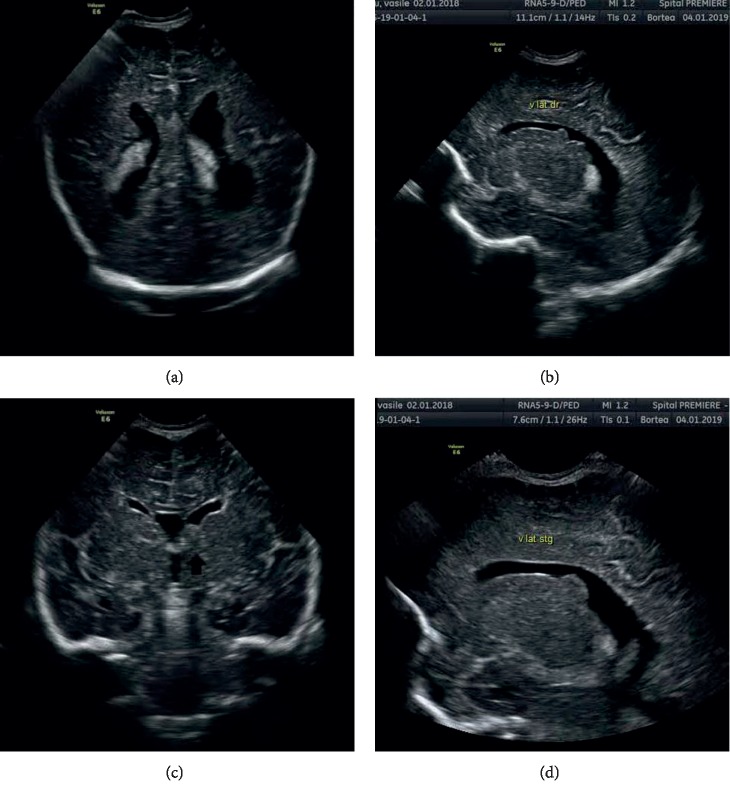
Cranial ultrasound—(a) bilateral hydrocephalus, mostly on the lest occipital horn-posterior coronal section; (b) well-delimitated, irregular, echogenic structures, bumping inside lateral ventricles—right parasagittal section; (c, d) echogenic structures near the left foramen of Monro, with a slight mass effect—coronal section (c) and left parasagittal section (d).

At birth, cranial ultrasound illustrated the bilateral increase of lateral ventricle, mostly on the left occipital horn. In the subependymal area were found the well-delimited, irregular, echogenic structures, some of them bumping inside lateral ventricles (Figure 2). The largest one was located near the left foramen of Monro (8/9 mm), with a slight mass effect. Echocardiography revealed a hyperechogenic well-marked structure, without significant ventricular prolapse, typical for rhabdomyoma, inside of each ventricular apex. Indirect ophthalmoscopy identified on the left eye retina two smooth, noncalcified, translucent lesions, next to the upper temporal vascular arcade. Neurological and electroencephalographic examinations and abdominal ultrasound were normal.

**Figure 3:**
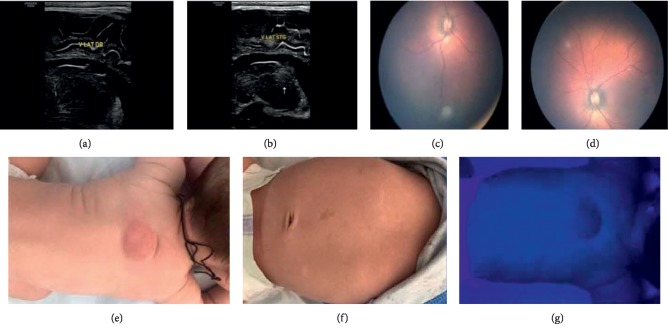
(a, b) Subependymal nodules, 8 weeks postnatal—right parasagittal section (a) and left parasagittal section (b); (c, d) multiple retinal hamartomas—indirect ophthalmoscopy; (e) Shagreen patch; (f) hypomelanotic macules—ash leaf; (g) hypomelanotic macules—confetti-like (Wood's lamp).

The postnatal ultrasound follow-up demonstrated a slight increase of hydrocephalus, mostly of occipital horn, with moderate asymmetry. At 8 weeks old, the first calcified, hyperechogenic area appeared next to the left foramen of Monro. Cardiac rhabdomyomas decreased in the right ventricular apex and could not be marked anymore in the left ventricular area. Indirect ophthalmoscopy monitoring registered multiple retinal hamartomas, two in the right eye and three in the left one (increasing in number). Dermatological examination, including Wood's lamp, pointed out size increase of Shagreen patch and appearing of hypomelanotic macules (ash leaf and confetti-like type) (Figure 3). Neurological symptoms occur at 7 weeks old with seizures-like subtle crises (infantile spasms). Vigabatrin was used as anticonvulsant treatment, with positive outcome.

Clinical diagnosis criteria from 2012 International Tuberous Sclerosis Complex Consensus Conference were used for diagnosis. Seven major criteria (hypomelanotic macules, Shagreen patch, multiple retinal hamartomas, cortical dysplasia, subependymal nodules, subependymal giant cell astrocytoma, and cardiac rhabdomyomas) and a minor one (“confetti” skin lesions) were taken into consideration.

The genetic diagnosis was done at 4 months old by sequencing analysis of the TSC1 and TSC2. A heterozygous likely pathogenic variant was identified in the TSC 2 gene—this finding is consistent with the genetic diagnosis of autosomal dominant tuberous sclerosis type.

## 3. Discussion

TSC patients present a high risk (70–80%) to develop epilepsy. Seizures usually appear during the first year of life, the onset being 4 to 6 months postnatal. Recent studies show that early seizures treatment of TSC patients could reduce their span and improve neurodevelopmental prognosis [6, 7]. In our case, the epileptic crises were diagnosed at 7 weeks old, and we introduced vigabatrin as treatment. As an outcome, the seizures were reduced in frequency and intensity. Vigabatrin is considered the first choice medication in treating TSC infantile spasms and can be regarded as a therapeutic approach in children with ictal discharged on EEG recordings, with or without clinical manifestation [7]. mTOR inhibitors can improve seizures outcome in drug-persistent epilepsy and are effective in reducing the volume of unamenable-to-surgery subependymal giant cell astrocytoma in TSC patients [5], but the safety of this treatment is not well studied at patients under the age of 1 year.

Fetal ultrasound screening can be used to find typical changes of TSC, most frequently cardiac rhabdomyoma, but is relatively limited when establishing the differential diagnosis [1]. In our case, the early diagnosis was possible because we identified the bilateral hydrocephalus at fetal ultrasound screening (32 w + 5 d). We decided that fetal MRI was necessary at that point because it supplements the information provided by ultrasound and leads to better inform counseling and management decisions [8, 9]. Its value is more useful after 24 weeks of gestation, and the main type of cerebral malformation in which fetal MRI played an important diagnostic role is space-occupying lesions [10]. The fetal MRI showed highly sensitive elements of TSC. The main changes found on fetal MRI in our patient are typical for TSC: subependymal nodules, subependymal giant cell astrocytoma, and transmantle cortical dysplasia. The last two changes were considered major risk factors for the early occurrence of seizures in our patient. It is important to consider the similar radiologic appearance of subependymal hemorrhage and nodules in antenatal MRI and the necessity of differential diagnosis [11].

In this case, fetal MRI provided a better pregnancy surveillance by identifying the cause of hydrocephalus even if this implied later on only ultrasound monitoring. The intrauterine diagnosis of TSC allowed us to start the seizures treatment by using the first choice medication for this disease, before having the genetic diagnosis confirmed.

Early diagnosis of TSC is possible and, in the light of the new studies and recommendations, it should be the central element in epilepsy management, with a better outcome. The fetal hydrocephalus diagnosed by pregnancy ultrasound screening must be investigated by fetal MRI. Neuroimaging (ultrasound and MRI), echocardiography, and Wood's lamp examination can support the diagnosis in perinatal-suspected TSC patients [12]. Considering the TSC neuropsychiatric impairment spectrum (mental retardation, autism, and ADHD), the early diagnosis is very important for its evolution and prognosis.
